# The Predominance of Ammonia-Oxidizing Archaea in an Oceanic Microbial Community Amended with Cyanobacterial Lysate

**DOI:** 10.1128/spectrum.02405-22

**Published:** 2023-01-09

**Authors:** Yufeng Jia, Madeline Lahm, Qi Chen, Leanne Powers, Michael Gonsior, Feng Chen

**Affiliations:** a Institute of Marine and Environmental Technology, University of Maryland Center for Environmental Science, Baltimore, Maryland, USA; b Chesapeake Biological Laboratory, University of Maryland Center for Environmental Science, Cambridge, Maryland, USA; c State Key Laboratory for Marine Environmental Science, Institute of Marine Microbes and Ecospheres, College of Ocean and Earth Sciences, Xiamen University, Xiamen, China; d Fujian Key Laboratory of Marine Carbon Sequestration, Xiamen University, Xiamen, China; e State University of New York College of Environmental Science and Forestry, Department of Chemistry, Syracuse, New York, USA; The Pennsylvania State University

**Keywords:** Thaumarchaeota, *Candidatus Nitrosopumilus*, oligotrophic ocean, *Synechococcus*-derived DOM, nitrogen cycling

## Abstract

When the oligotrophic microbial community was amended with *Synechococcus*-derived dissolved organic matter (SDOM) and incubated under the dark condition, archaea relative abundance was initially very low but made up more than 60% of the prokaryotic community on day 60, and remained dominant for at least 9 months. The archaeal sequences were dominated by *Candidatus Nitrosopumilus*, the Group I.1a Thaumarchaeota. The increase of Thaumarchaeota in the dark incubation corresponded to the period of delayed ammonium oxidation upon an initially steady increase in ammonia, supporting the remarkable competency of Thaumarchaeota in energy utilization and fixation of inorganic carbon in the ocean.

**IMPORTANCE** Thaumarchaeota, which are ammonia-oxidizing archaea (AOA), are mainly chemolithoautotrophs that can fix inorganic carbon to produce organic matter in the dark. Their distinctive physiological traits and high abundance in the water column indicate the significant ecological roles they play in the open ocean. In our study, we found predominant Thaumarchaeota in the microbial community amended with cyanobacteria-derived lysate under the dark condition. Furthermore, Thaumarchaeota remained dominant in the microbial community even after 1 year of incubation. Through the ammonification process, dissolved organic matter (DOM) from cyanobacterial lysate was converted to ammonium which was used as an energy source for Thaumarchaeota to fix inorganic carbon into biomass. Our study further advocates the important roles of Thaumarchaeota in the ocean’s biogeochemical cycle.

## OBSERVATION

Microorganisms play an important role in the transformation of dissolved organic matter (DOM) in the ocean. Marine DOM is one of Earth's largest carbon reservoirs and plays a major role in the global carbon cycle (1). A fraction of the DOM pool can persist in the deeper ocean (2), but how long DOM is sequestered remains under debate. Hence, studies that shed light on the reactivity of deep-ocean DOM are needed. To understand the interaction between microorganisms and chemical compounds, laboratory incubation experiments are usually conducted by adding known sources of DOM into natural microbial communities (3, 4) and evaluating its transformation or mineralization. Picocyanobacteria are major contributors to primary production in the ocean (5), and DOM derived from picocyanobacteria contributes greatly to the ocean's DOM pool (6). Picocyanobacterial lysate has been used as a source of organic matter to study microbial response in laboratory settings, and the bacterial community responds actively to the addition of picocyanobacterial DOM (7, 8). However, very little is known about the role of the archaeal community in such *in vitro* incubation systems. A recent study found the enrichment of the archaeal community on day 20 after cyanobacterial lysate was added into the coastal water (8). In this study, we report the predominance of archaea (Thaumarchaeota) in the later stage of the *in vitro* incubation where oligotrophic microbial communities were amended with cyanobacterial lysate.

In our incubation system, *Synechococcus*-derived DOM (SDOM) was amended to the seawater collected from the surface of the Gulf Stream (34° 9′ 33.73″ N, 77° 43′ 57.73″ W) to monitor the change of the prokaryotic community during the *in vitro* incubation. The lysate of *Synechococcus* cells (open ocean strain WH7803) was prepared using French press (Glenn Mills, Clifton, NJ, USA), and filtered through a precombusted 0.7 μm GF/F filter (Whatman, Maidstone, UK) to collect the DOM fraction. SDOM (50 mL) was added to 20 L of the oceanic water, and both treatments (with SDOM, *n* = 3) and controls (without SDOM, *n* = 3) were incubated in the dark at room temperature (22°C). More methodological details, including subsample collection, DNA extraction, PCR amplification, sequencing, bioinformatic analysis, and water chemical analysis, are described in the supplemental online material.

**FIG 1:**
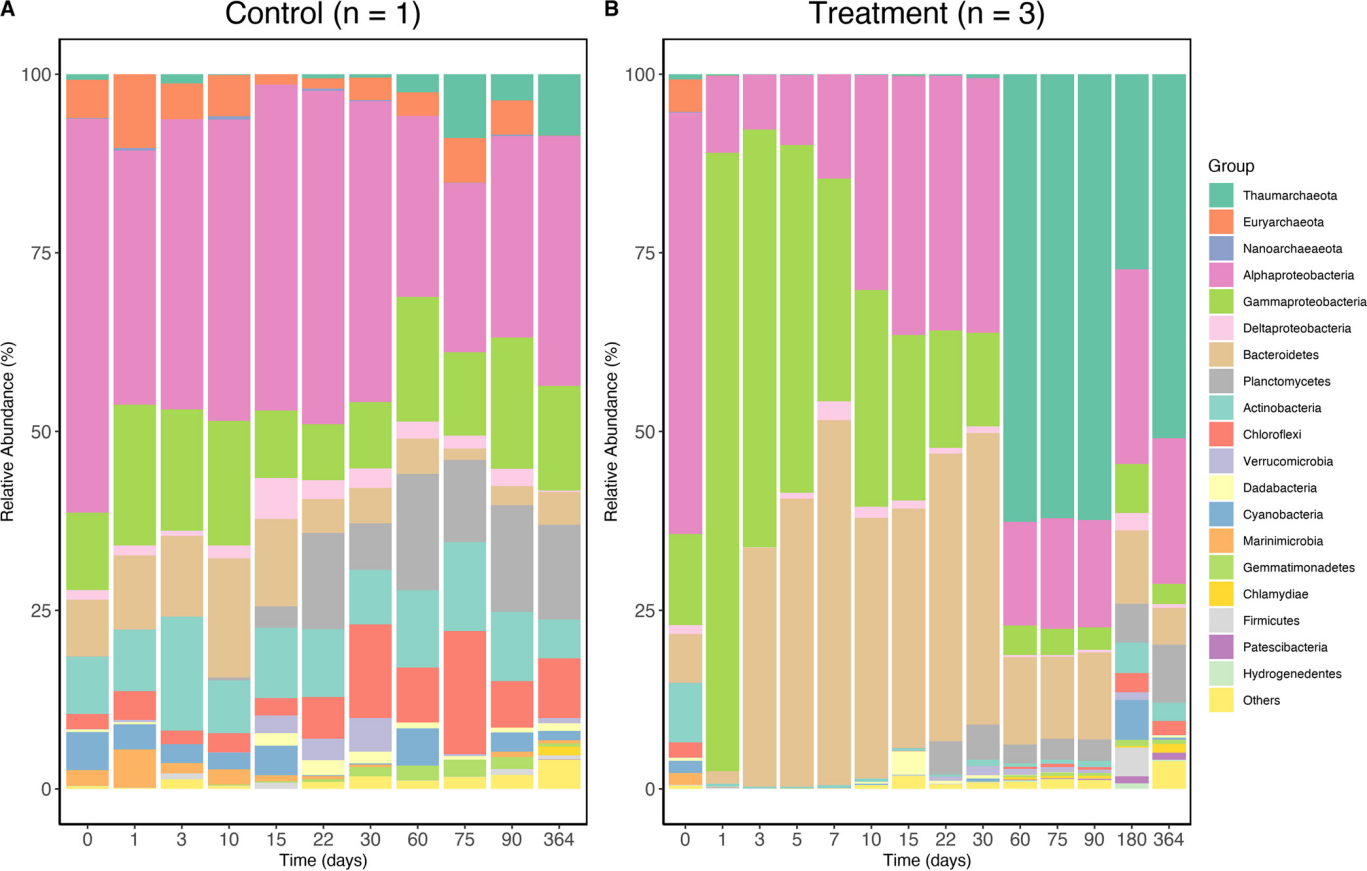
The relative abundance of the top 20 bacterial and archaeal groups in the microbial community at different sampling time points (day 0 to day 364) of the incubation experiment. Microbial community compositions are shown for the control (A) and the SDOM treatment (B). Relative abundance values for the treatment are averages of triplicates. For the control, due to the low microbial biomass of oceanic water, DNA from triplicate samples were combined in order to generate positive PCR. Three control subsamples (day 5, 7, and 180) were not able to generate positive PCR.

**FIG 2:**
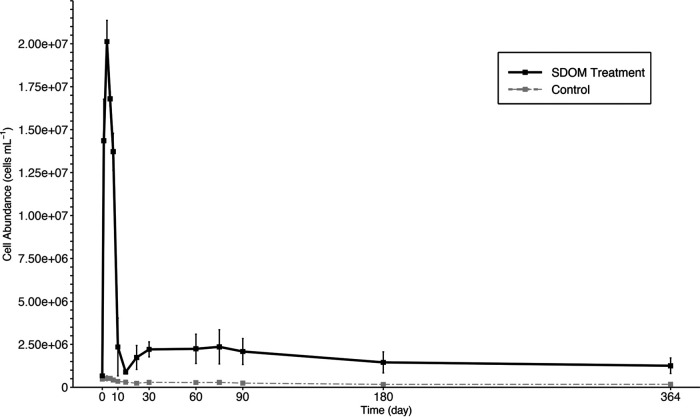
Microbial cell abundance during the incubation experiment for the *Synechococcus*-derived DOM treatment (black) and the seawater control (gray). Error bars indicate the standard deviations of triplicates of the treatment group.

A clear shift of bacterial community upon the addition of SDOM was evident (Fig. 1), but what was intriguing was the unexpected occurrence of highly abundant archaea in the later stage of incubation. On day 60, Thaumarchaeota emerged and made up 62% of the prokaryotic community in the treatment, and the predominance of Thaumarchaeota persisted from day 60 to 364 (Fig. 1B). In contrast, Thaumarchaeota only represented up to 8% of the community in the control during the same period (Fig. 1A). Flow cytometric analysis showed that the microbial cell abundance was relatively stable for the treatment after day 10 (Fig. 2). Therefore, the change in microbial cell abundance hardly contributed to the dramatic shift in the relative abundance of Thaumarchaeota in the later stage. Thaumarchaeota in the treatment made up the majority of the prokaryotic community at the later stage of incubation, and this was a dramatic increase since they only accounted for a very small fraction (0.8%) of the prokaryotic community in the original water (day 0). Almost all the Thaumarchaeota sequences were classified as *Candidatus Nitrosopumilus*, which belongs to group I.1a of the Thaumarchaeota lineage (9). Group I.1a Thaumarchaeota are ubiquitous in the oligotrophic ocean, and they are dominant ammonia-oxidizing archaea (AOA) in the water column (10, 11). In the treatment, the concentration of ammonium increased sharply in the first 10 days, which was supplied by ammonification, as dissolved organic matter (carbon, nitrogen, phosphorus) sharply decreased in concentration (Fig. S1). The concentration of ammonium remained high from day 10 to 30, before quickly decreasing to the control level during day 30 and 60 (Fig. S1B), likely oxidized to nitrite by the mesophilic marine Thaumarchaeota (12, 13). The delayed predominance of Thaumarchaeota after more than 20 days of high ammonia concentration may be due to their slow growth rate.

It is plausible that Thaumarchaeota were responsible for the first step of nitrification (ammonium to nitrite) in the incubation as their relative abundance increased from 0.5% to 62% during this period (Fig. 1B). Thaumarchaeota generally have higher ammonium affinities compared to ammonium oxidizing bacteria (AOB) (14, 15), and such a feature may explain the persistence of Thaumarchaeota during day 60 and 364 when the concentration of ammonium was low. Thaumarchaeota strains have been reported to be adapted to oligotrophic conditions with low ammonium concentration (in the nM to μM range) (15).

The occurrence of high abundant Thaumarchaeota in the late stage suggests a potential niche preferable to the AOA community after prolonged dark incubation. This phenomenon could be missed in earlier studies if the PCR primers only target bacterial communities or the incubation time is not long enough (7, 16). Moreover, a study has shown that the microbial community tends to resemble the original assemblage in the late stage of incubation (i.e., 60 to 90 days) (7). This claim may not hold when the predominance of archaea persists as our study shows.

In an earlier study where a coastal microbial community was amended with SDOM, Thaumarchaeota became dominant on day 20 (~52%) and decreased to ~20% from day 80 to 180 (8). It appears that Thaumarchaeota can become more dominant and persist longer when the open ocean water was amended with SDOM. It is intriguing that the relative abundance of Thaumarchaeota remained rather stable (~62%) between day 60 and 364. The microbial community in our study was collected from the oligotrophic open ocean, which selects different microbial assemblages compared to the coastal waters with higher nutrients. Could the varied compositions of the initial coastal and oceanic microbial communities play a role in the different responses of Thaumarchaeota? Another potential cause is the difference in the composition of cyanobacterial lysate. The previous study added the whole cyanobacterial lysate which includes both DOM and particulate organic matter (POM) (8), while we only added the DOM fraction in our study.

The marine photic zone is dominated by photoautotrophs like picocyanobacteria and other phytoplankton. AOA are chemolithoautotrophs, which can fix bicarbonate to produce organic matter in the dark, as genomic studies have shown their carbon assimilation pathways (17). AOA physiology and diversity in the ocean have been extensively studied since mesophilic Thaumarchaeota are abundant throughout the water column (13, 18, 19). Thaumarchaeota are well adapted to environments with low or no light, low nutrients, and low dissolved oxygen (10, 15), and they were considered to significantly participate in dark primary production (20). AOA oxidize ammonia provided by the ammonification of DON from primary and secondary producers to garner the energy required for inorganic carbon fixation. Our study confirms the AOA dominance in the microbial assemblages during the pulse of cyanobacteria-derived DOM using *in vitro* approach and emphasizes the crucial roles of AOA in the biogeochemical cycles in the open ocean.

### Data availability.

Sequence data were deposited in the National Center for Biotechnology Information (NCBI) Sequence Read Archive with BioProject PRJNA906266.
