# Supplementary material for: The Predominance of Ammonia-Oxidizing Archaea in an Oceanic Microbial Community Amended with Cyanobacterial Lysate
Source: Microbiol Spectr. 2023 Jan 9;11(1):e02405-22. doi: 10.1128/spectrum.02405-22 (PMC9927567; doi:10.1128/spectrum.02405-22)
Supplement: Supplemental file 1 — Fig. S1 and supplemental text. Download spectrum.02405-22-s0001.pdf, PDF file, 0.2 MB [file spectrum.02405-22-s0001.pdf]

Supplemental Information

Figure S1. Variations of DOM and nutrients in the treatment (solid lines) and seawater control (dashed lines) during the 364-day incubation. The chemicals shown here are: (A) dissolved organic carbon (DOC); (B) dissolved organic nitrogen (DON), ammonium, nitrite, and nitrate; (C) dissolved organic phosphorus (DOP) and phosphate. Error bars indicate standard deviation calculated from triplicate samples.

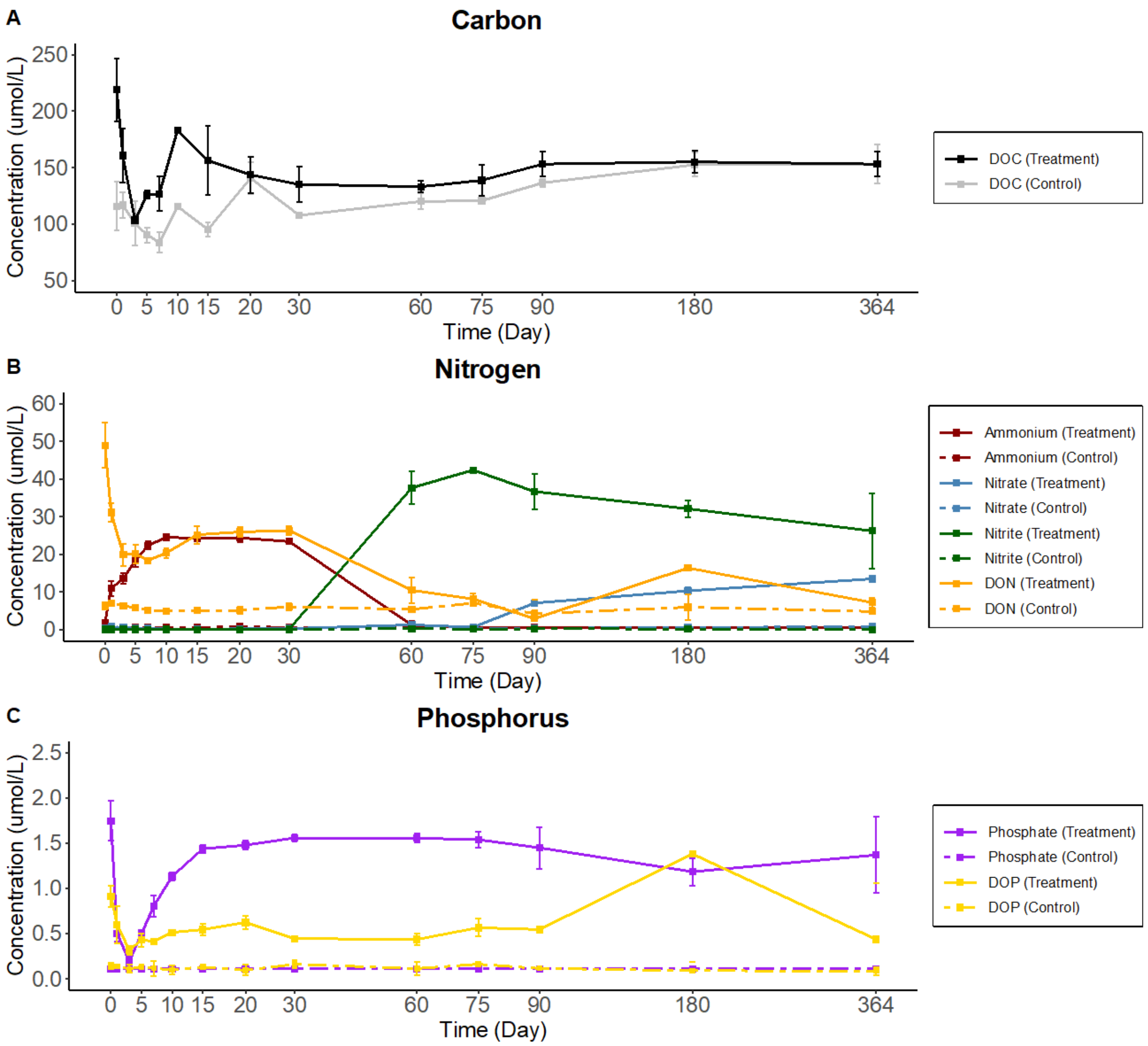
A
Carbon
Concentration (umol/L)
250
200
150
100
50
0 5 10 15 20 30 60 75 90 180 364
Time (Day)
DOC (Treatment)
DOC (Control)
B
Nitrogen
Concentration (umol/L)
60
50
40
30
20
10
0
0 5 10 15 20 30 60 75 90 180 364
Time (Day)
Ammonium (Treatment)
Ammonium (Control)
Nitrate (Treatment)
Nitrate (Control)
Nitrite (Treatment)
Nitrite (Control)
DON (Treatment)
DON (Control)
C
Phosphorus
Concentration (umol/L)
2.5
2.0
1.5
1.0
0.5
0.0
0 5 10 15 20 30 60 75 90 180 364
Time (Day)
Phosphate (Treatment)
Phosphate (Control)
DOP (Treatment)
DOP (Control)

Fig. S1

Subsample collection

Following incubation preparation, subsamples of 350 mL were collected on day 0, 1, 3, 5, 7, 10, 15, 22, 30, 60, 75, 90, 180, and 364 from both SDOM treatments (n = 3) and controls (n = 3). Of the 350 mL subsample, 300 mL was filtered through a 0.22µm polycarbonate filter and stored at -20 ˚C for subsequent DNA analysis. The filtrate from the same filter was stored at 4 ˚C for nutrient analysis. The other 50 mL were filtered with a pre-combusted Whatman® 0.7 µm GF/F filter and spiked with 50 µL 32% HCl for dissolved organic carbon (DOC) measurement.

Prokaryotic community analysis

Microbial DNA was extracted from 0.22 µm filters, using a modified phenol-chloroform protocol [1]. Briefly, thawed filter was placed in a Whirl-Pak bag, and 2 mL lysis buffer (0.1 M Tris-HCl, pH = 8.0; 0.1 M EDTA; 0.8 M sucrose) and 10 µL lysozyme (200 µg/µL) were added into the bag. The Whirl-Pak bag was incubated at 37 ˚C for 30 min, amended with 10 µL proteinase K (20 mg/mL) and 10 µL SDS (final concentration 1%), and further incubated at 37 ˚C overnight [2]. The solution was added 100 µL CTAB + NaCl (10%, 1.4 M) and incubated at 65 ˚C for 30 min. DNA was partitioned with phenol:chloroform:isoamyl alcohol (25:24:1, v/v) and chloroform:isoamyl alcohol (24:1, v/v) via centrifugation, and then precipitated by isopropanol. DNA precipitate was washed with cold ethanol (4˚C, 70%) and eluted with nuclease-free water.

The V4 region of microbial 16S rRNA gene was amplified with PCR using forward primer 515F (5'-GTGYCAGCMGCCGCGGTAA-3') [3] and reverse primer 806R (5'-GGACTACNVGGGTWTCTAAT-3') [4]. High-throughput DNA sequencing was performed on the Illumina MiSeq platform, and the raw sequences were treated using the QIIME 2 (version

2020.2) pipeline [5]. Operational taxonomic units (OTUs) were generated from raw reads by quality trimming, Dada2 denoising, and clustering, and their taxonomy were classified with SILVA database [6] by a machine learning software plugin scikit-learn [7].

Water chemical analysis

Chemical measurements were conducted following the same methods by Zhao et al. [8]. Briefly, DOC and total dissolved nitrogen (TDN) were analyzed using a Shimadzu TOC-V and aTNM-1 unit. All samples were run in triplicates and then averaged. Dissolved inorganic orthophosphate (PO_4^{3-}) was measured using EPA method 365.1 and the reaction of phosphate with ammonium molybdate and potassium antimony tartrate to form a blue-colored complex. Inorganic ammonium was quantified using the Bertholet reaction and the formation of a blue-colored compound after its reaction with sodium phenoxide. Nitrite and nitrate were measured using EPA method 353.2 and cadmium reduction of nitrate to nitrite. Nitrite is then quantified colormetrically upon its reaction with sulfanilamide and coupling to N-1-napthylethylenediamine dihydrochloride to form an azo dye. Concentration of dissolved organic nitrogen (DON) was calculated by subtracting inorganic nitrogen (NH^{4+}, NO^{3-} and NO^{2-}) from TDN.

References

1. Kan J. 2006. Bacterioplankton in the Chesapeake Bay: genetic diversity, population dynamics, and community proteomics. Doctoral dissertation. University of Maryland, College Park, MD, USA.

2. Kan J, Wang K, Chen F. 2006. Temporal variation and detection limit of an estuarine bacterioplankton community analyzed by denaturing gradient gel electrophoresis (DGGE). *Aquat Microb Ecol* 42:7-18.

3. Parada AE, Needham DM, Fuhrman JA. 2016. Every base matters: Assessing small subunit rRNA primers for marine microbiomes with mock communities, time series and global field samples. *Environ Microbiol* 18:1403–1414.

4. Apprill A, Mcnally S, Parsons R, Weber L. 2015. Minor revision to V4 region SSU rRNA 806R gene primer greatly increases detection of SAR11 bacterioplankton. *Aquat Microb Ecol* 75:129–137.

5. Bolyen E, Rideout JR, Dillon MR, Bokulich NA, Abnet CC, Al-Ghalith GA, Alexander H, Alm EJ, Arumugam M, Asnicar F, Bai Y, Bisanz JE, Bittinger K, Brejnrod A, Brislawn CJ, Brown CT, Callahan BJ, Caraballo-Rodríguez AM, Chase J, Cope EK, Da Silva R, Diener C, Dorrestein PC, Douglas GM, Durall DM, Duvallet C, Edwardson CF, Ernst M, Estaki M, Fouquier J, Gauglitz JM, Gibbons SM, Gibson DL, Gonzalez A, Gorlick K, Guo J, Hillmann B, Holmes S, Holste H, Huttenhower C, Huttley GA, Janssen S, Jarmusch AK, Jiang L, Kaehler BD, Kang KB, Keefe CR, Keim P, Kelley ST, Knights D, Koester I, Kosciolek T, Kreps J, Langille MGI, Lee J, Ley R, Liu Y, Loftfield E, Lozupone C, Maher M, Marotz C, Martin BD, McDonald D, McIver LJ, Melnik AV, Metcalf JL, Morgan SC, Morton JT, Naimey AT, Navas-Molina JA, Nothias LF, Orchanian SB, Pearson T, Peoples SL, Petras D, Preuss ML, Pruesse E,

Rasmussen LB, Rivers A, Robeson II MS, Rosenthal P, Segata N, Shaffer M, Shiffer A, Sinha R, Song SJ, Spear JR, Swafford AD, Thompson LR, Torres PJ, Trinh P, Tripathi A, Turnbaugh PJ Ul-Hasan S, vander Hooft JJJ, Vargas F, Vázquez-Baeza Y, Vogtmann E, von Hippel M, Walters W, Wan Y, Wang M, Warren J, Weber KC, Williamson CHD, Willis AD, Xu ZZ, Zaneveld JR, Zhang Y, Zhu Q, Knight R, Caporaso JG. 2019. Reproducible, interactive, scalable and extensible microbiome data science using QIIME 2. *Nat Biotechnol* 37:852–857.

6. Quast C, Pruesse E, Yilmaz P, Gerken J, Schweer T, Yarza P, Peplies J, Glöckner FO. 2013. The SILVA ribosomal RNA gene database project: improved data processing and web-based tools. *Nucleic Acids Res* 41:D590-D596.

7. Pedregosa F, Varoquaux G, Gramfort A, Michel V, Thirion B, Grisel O, Blondel M, Prettenhofer P, Weiss R, Dugourg V, Vanderplas J, Passos A, Cournapeau D, Brucher M, Perrot M, Duchesnay É. 2011. Scikit-learn: machine learning in Python. *J Mach Learn Res* 12:2825-2830.

8. Zhao Z, Gonsior M, Schmitt-Kopplin P, Zhan Y, Zhang R, Jiao N, Chen F. 2019. Microbial transformation of virus-induced dissolved organic matter from picocyanobacteria: coupling of bacterial diversity and DOM chemodiversity. *ISME J* 13:2551–2565.
